# Time to care? Health of informal older carers and time spent on health related activities: an Australian survey

**DOI:** 10.1186/1471-2458-13-374

**Published:** 2013-04-22

**Authors:** Tanisha Jowsey, Ian McRae, James Gillespie, Michelle Banfield, Laurann Yen

**Affiliations:** 1 Australian Primary Health Care Research Institute, Australian National University, Canberra, ACT, 0200, Australia; 2 Menzies Centre for Health Policy, University of Sydney, Sydney, NSW, 2006, Australia; 3 Menzies Centre for Health Policy, University of Sydney, Sydney, Australia

**Keywords:** Carer, Informal care, Health services, Respite, Chronic illness, Long term condition, Chronic disease, Time, Time use, Australia, Health related activities

## Abstract

**Background:**

Little is known about the time spent on specific health related activities by older adult informal carers who assist people with chronic illness. Research has not yet addressed the association between carer health status and their care demands. Such information could inform policy and health system efforts to manage chronic illness.

**Methods:**

We conducted an Australia wide survey using recall questionnaires to record time use. The study asked how much time is spent on “most days” for the most common activities like taking medication, self-treatment and testing, and how much time in the last month on less common activities like attending a physician or shopping associated with health needs. The survey was mailed to 5,000 members of National Seniors Australia; 2,500 registrants on the National Diabetes Services Scheme; and 3,100 members of the Australian Lung Foundation. A total of 2519 people responded, including 313 people who identified as informal carers. Statistical analysis was undertaken using Stata 11. Standard errors and confidence intervals were derived using bootstrapping techniques within Stata 11.

**Results:**

Most carers (96.2%) had chronic illness themselves, and those with greater numbers of chronic illnesses were those who faced the greatest overall time demands. The top decile of carers devoted between 8.5 and 10 hours a day to personal and caring health related activities. Informal carers with chronic illness spent more time managing their own health than people with chronic illness who were not informal carers. These carers spent more time on caring for others than on caring for their own health. High levels of caring responsibility were associated with poorer reported carer health.

**Conclusions:**

Policy and health care services will need to adapt to recognise and reduce the time burden on carers who themselves have chronic illness. More carefully targeted investment in the social infrastructure of formal care would free up carers for other activities (including their own care) and holds the potential to improve the quality of life as well as the health outcomes of this population.

## Background

It is no secret that ageing populations and rapidly increasing rates of chronic illness are creating unprecedented pressure on health and social support systems in all industrialised countries, often exacerbated by healthcare workforce insufficiencies. Health policy responses have included, among other things, increased discourse and support for patient self-management [1,2]; which in practice often implicates family members and friends (‘carers’ hereafter) of people with chronic illness [3-5]. Carers become a key source of care, supported, as the functional ability of the care recipient decreases, by a range of visiting services such as the Home Care Support Program in Canada [6] and the Home and Community Care Program in Australia [7].

Australian and international studies have suggested that caring activities can be very demanding and can adversely affect carer health [3,8]. Higher levels of carer burden are matched by lower self-perceived health status than non-carers and increased prevalence of cognitive impairment [9]. Informal caring can be stressful [3,10,11] and can lead to deterioration in physical and psychological health [12-16]. In the United States, Miller and colleagues have observed that carers “must also cope with their own health problems, typically exacerbated by their caregiving responsibilities” [17]. Nonetheless, carers are relied upon to provide substantial levels of unpaid support including to those with chronic illness.

Creating support strategies for carers relies on understanding the work they do and their areas of need [1,18,19]. In our earlier qualitative study people living with chronic illness reported the types of care activities undertaken and they suggested that further health service and policy support, including carer respite, would help [3]. That study did not measure the quantum of caring tasks undertaken. Informal care activities usually include health and personal services, household chores, running errands, and providing emotional, social and psychological support as well as the organisation and transport to formal care appointments [3,20].

The components of carers’ work have been studied only in broad terms in Australia and elsewhere (see for example, [21]). Such studies have reported that carers spend considerable amounts of time dedicated to caring activities but studies have not sought to differentiate between the care activities provided for care recipients with chronic illness from those with other functional impairments. Australian surveys of informal care have told us little about the component activities of time spent caring, and in particular about time spent on specific health related activities (HRA). For example, Bittman and Thomson’s [22] Australian study of informal carer time use (which includes chronic illness care as well as parent care of children with mental and physical impairments), reported that an average of around five hours per week was spent in care-related activities. A subsequent study, which adjusted the estimates from the 1997 Australian Time Use Survey, found that the median time spent by informal carers is around an hour and a half per day (or 10.5 hours per week) [23]. Bittman and colleagues also reported that carers of people with a functional impairment (including but not limited to impairments related to chronic illness) spend considerably more time engaging in cooking and cleaning activities than non-carers [24]. However, they give no specific information about the time spent on other care-related activities.

As with the Australian studies reported above, time use studies in North America have shown that while some people experienced high time burdens associated with providing care, the median time spent was 78–115 minutes a day [25,26]. However these studies do not report which people might face the greatest time demands nor the magnitude of those time demands. Finally the literature does not report the health status or the care needs of carers and whether their own chronic illness means they spend more time on caring than carers without this extra burden. We suggest that gaps remain in knowledge about the daily ‘work’ of caring for someone with a chronic illness [13,27], in particular when the carer has their own health problems to manage.

This study addresses some of these gaps. We use survey findings from an Australian study of older adults to explore the time spent on health related activity (HRA), as distinct from normal household activity, that is associated both with caring for another and with self-management. We suggest that HRA comprises the work of managing illness that is additional to normal activities of daily living; such as navigation and interaction with health care services, managing medications, and maintaining a healthy diet [27]. We report how much work carers undertake by both activity type and time.

First, in order to measure the work of caring for people with chronic illness we explore the composition of health related caring and self-management activity. Second, we examine time spent on HRA by people with chronic illness, both in relation to managing their own health and in relation to managing the health of someone they care for. The relationship between time spent on own health and on caring is detailed. We also examine some of the details of the time spent on specific HRA to observe where the greatest demands lie, and further look at those who spend the most time on caring-HRA to examine how great the demands on these groups of people may be.

## Methods

A mail survey was carried out; “How much work is involved in looking after your health?” It built on an earlier qualitative study of people, both patients and carers, living with chronic illness in the western suburbs of Sydney and the Australian Capital Territory [28].

### Sample design

The sample was drawn from three sub-populations of older Australians. National Seniors Australia (NSA) is a member organisation of Australians aged 50 and over with 285,000 members from which a sample of 5,000 people was drawn. NSA broadly represents the older Australian community [29], and does not contain a large number of people who are seriously ill. To increase the sample of people with more severe chronic illness, older people were oversampled from this population. Further samples were drawn from the National Diabetes Services Scheme (NDSS), a government funded service which provides subsidies for diabetes materials with 280,000 of its registrants aged over 50 years (sample size 2,500) and the Lung Foundation Australia (LFA), a member organisation which supports research into lung conditions and provides member support (sample of all 3,109 persons with chronic obstructive pulmonary disease (COPD) or who supported a person with COPD. Almost all people in this group were aged over 50 years). For ease of reading we use the terms ‘Lung sub-sample’ to reference the LFA sample, and ‘Diabetes sub-sample’ to reference the NDSS sample.

### Questionnaire design

Time use was defined as the time reportedly spent on any of three HRA:

1. Activities related to use of medical and allied health services in the previous month; such as making appointments, travelling to health services, waiting in waiting rooms, attending appointments and having medical treatments. These activities are referred to as ‘clinic activities’.

2. Activities related to obtaining information, support or products in the previous month; including attending rehabilitation programs, education programs and support groups, shopping for special foods and looking for/reading health information. These activities are referred to as ‘other activities’.

3. Activities undertaken in domestic spaces on most days (such as time spent on exercising, preparing/consuming prescribed medications, and undertaking tests at home such as blood glucose monitoring). These activities are referred to as ‘home activities’.

The questionnaire also collected demographic data (including age, gender, indigenous status, region of birth, whether speaking English at home, postcode, number in household, household income, marital status, employment status, and highest qualification); and self-reported use of health services. The socio-economic status of each respondent was indicated by the Index of Relative Socio-Economic Disadvantage for the postcode in which they lived, which was drawn from the ABS Census 2006 [30].

Two standard measures of self-assessed health were included in the survey (the SF12 and EQ5D). Respondents provided information about the time spent on HRA in relation to their own health, and then on HRA related to the health of the person/persons for whom they cared. The two sets of questions were aligned to allow comparison of caring and own health activities.

The measurement of time use in informal care has relied on either diaries (see for example, [23]) or recall (see for example, [26,31]). While keeping diaries is often regarded as the most accurate data collection method, it is both expensive and potentially intrusive. Recall questionnaires were used in this study to limit the burden of research participation on the respondents, to encourage response, and to provide data which covered longer periods, increasing event numbers and accessing long term rather than daily distribution of time use [23,26,31].

Questions on time use (for both own health and caring) asked how much time was spent “on most days” for regular tasks such as managing and taking medication; how much over the last month for less regular activities that included attending rehabilitation programs, shopping for special foods, seeking information; and how much on health service linked activities such as travelling to, waiting for and attending a doctor (see Attachment A1).

The questionnaire drew on questionnaires previously tested by McRae and colleagues [4,29] and international time use surveys from North America [25] and Australia [23]. The questionnaire was piloted with 18 members of a local health service consumer network. They suggested changing some terminology, simplifying questions and shortening the survey, which we did. The revised survey was re-tested by 28 older Australians who had taken part in an earlier survey and indicated their willingness to participate in further research. No further changes were made as a result. The revised survey was mailed to selected individuals (as below), with the option to complete it on line using Survey Monkey®, a proprietary survey tool, or to complete the form and return it by prepaid post.

Ethics approval for the survey was obtained from the Australian National University Human Research Ethics Committee (Protocol number: 2010/468) in 2010.

### Analysis

Survey responses were computer coded and entered into STATA 11 [32]. Carers were identified by their completion of the carer section of the survey form, and their responses analysed. The results are presented in terms of descriptive statistics addressing various questions, and were estimated using STATA 11 [32]. Time use is presented in terms of hours per month on each activity. As the distribution of time use is highly skewed, results are presented using medians. To address issues of the highest time demands the 90^th^ percentiles are presented.

To enable comparison with previous studies which address the question of whether carers with low levels of caring activity are healthier than those who do no caring we compare the proportions of carers and non-carers with poor or fair health. We use the carers in the lowest quartile of caring time as those with “low levels of caring”, following Buyck et al’s use of the lowest quartile [9].

Respondents were asked how much time they spent on exercise and physical activity each day. Because exercise time tended to dominate all other reported times, it has been reported separately to allow the less time consuming activities to emerge. While the majority of respondents spent some time on HRA, many people did not spend time on particular health related activities included in the survey (e.g. attending rehabilitation, preparing special foods). When looking at the more detailed time components we therefore report on both the proportion of people undertaking each task, and time spent by those undertaking them. Estimates were weighted to stratum populations for each sub-sample. Given their different structures and different population sizes the results for three samples are reported separately. Standard errors and confidence intervals were derived using bootstrapping techniques within Stata 11.

## Results

Response rates differed between the three sub-samples with an overall response of 2,540 (24.0%). Most respondents returned the completed printed survey, with only 75 respondents completing the survey online (uploaded through Survey Monkey). Response rates were highest in the NSA sub-sample (28.6%) which best represents the health status of the overall older population, and lowest in the sub-sample of those registered with NDSS, all diagnosed with diabetes mellitus (17.1%). The response rate for the Lung sub-sample was 24.0%. Broadly the youngest and oldest were least likely to respond, there was little difference in male and female response rates, and while there were differences in response rates by States there were no obvious patterns across the three sub-sample groups.

### Structure of the respondent populations

Overall 12.4% (N = 313) of respondents identified themselves as caring for other people and provided estimates of time spent on caring. Of those carers, 62% cared for a partner/spouse. All but 12 carer respondents had at least one chronic illness. The carers looking after parents were, on average, almost three years younger than those looking after spouses. Those caring for spouses were estimated to spend more time than those caring for parents. However, the differences were not statistically significant. Thirty of the 313 carers (9.5%) did not report a chronic illness for their care recipient, so the people cared for are assumed to have had a disability derived from some other source.

Table 1 shows the sample size and basic demographics for carers and non-carers with and without their own chronic illness in each sub-sample. Within the NSA sub-sample the most prevalent reported chronic illnesses include arthritis (49.9%), depression/anxiety (32.5%), cancer (23.6%) and chronic pain (21.2%). In addition to diabetes, the most prevalent co-morbid illnesses that respondents in the Diabetes sub-sample reported include chronic pain (48.1%), arthritis (43.4%), and depression/anxiety (42.8%). In addition to COPD, the most prevalent co-morbid illnesses that respondents in the Lung sub-sample reported include depression/anxiety (38.8%), asthma (36.7%), arthritis (33.1%) and chronic pain (32.9%). Chronic pain and depression/anxiety were thus, among the most commonly reported illnesses in all three sub-samples. The illnesses associated with self-reported highest time use include respiratory diseases and diabetes.

**Table 1:** Sample responses and estimated sample characteristics

	**Diabetes sub-sample**		**Lung sub-sample**		**NSA subsample**			
	Carer with chronic condition	Not a carer with chronic condition	Carer with chronic condition	Not a carer with chronic condition	Carer with chronic condition	Not a carer with chronic condition	Carer with no chronic condition	Not a carer with no chronic condition
Sample*	55	368	117	559	130	1,107	12	183
Estimated population structure								
% Male	47.3%	57.4%	52.4%	39.4%	35.6%	40.2%	35.0%	41.4%
% aged <60 years	19.4%	26.1%	9.8%	11.7%	26.3%	25.6%	45.6%	33.3%
% aged 60–69 years	30.6%	36.5%	33.1%	34.6%	43.4%	49.3%	50.1%	54.6%
% aged 70 years or older	50.0%	37.4%	57.1%	53.7%	30.4%	25.1%	4.3%	12.1%
% caring for parent	34.1%		16.8%		21.3%		31.4%	
% caring for partner	60.3%		73.6%		62.9%		52.4%	
% caring for son/daughter	1.2%		1.2%		5.9%		16.2%	
% caring for other	4.5%		8.5%		9.9%		0.0%	

*Note: in theory we expected no respondents from the DBT and LNG samples without chronic conditions, but have found 4 and 5 respectively. These may be people who joined the bodies to support their family members.

### Health of carers and non-carers

Carers were more likely to report poorer health than non-carers. Table 2 shows the differences between carers and non-carers reporting poor or fair self-assessed health. In all cases the differences between carers and non-carers are significant and material (p = 0.000, 0.012, 0.003 for diabetes, lung and NSA sub-samples respectively). Table 2 also shows the differences in self-assessed health status between those in the lowest quartile of caring time (less than 13 hours per month) and those in the highest quartile. None of these differences is significant, and the diabetes and lung sub-samples are in the opposite direction to the NSA sub-sample. The self-assessed health of the least active carers is still worse than that of the non-carers, although the differences are only significant in the case of the diabetes sub-sample. If adjustment is made for the age, gender and social status of the carers, the pattern of those providing low levels of care reporting poorer self-assessed health remains, but is again not significant.

**Table 2:** Health of carers and non carers

	**Diabetes sub-sample**	**Lung sub-sample**	**NSA sub-sample**
**Percent with poor or fair self-assessed health (95% CI)**			
Carers in upper three quartiles of caring time	62.3% (47.1-77.5)	80.3% (71.9-88.6)	27.6% (20.0-35.1)
Carers in lowest quartile of caring time	67.5% (43.9-91.1)	82.87% (70.4-95.2)	18.3% (8.5-28.1)
Total Carers	63.2% (50.7-75.7)	80.5% (72.9-88.2)	24.8% (18.6-31.0)
Non-carers	35.0% (30.2-39.8)	70.5% (65.9-75.0)	15.9% (13.9-18.0)

### Time spent on own care and on caring

The total time respondents reported spending on their own personal and caring HRA was related to whether the respondent was a carer and whether they had a chronic illness. As shown in Table 3, carers spent more time on their own health than non-carers (consistent with the health status reported in Table 2). For the NSA and Lung sub-samples the differences between carers with chronic illnesses and non-carers with chronic illnesses were significant. In the NSA sub-sample where the differences between those with and without chronic illnesses can be observed, these differences in time spent on their own health were significant for both carer and non-carer groups. Time spent caring, on the other hand, was not significantly different depending on whether carers had chronic illnesses.

**Table 3:** Median time spent on HRA (hours per month, 95% CI in parentheses)

	**Diabetes sub-sample**	**Lung sub-sample**	**NSA sub-sample**
**Time spent on own health**			
Not a carer with chronic condition	10.5 (8.9-12.1)	15.7 (14.1-17.2)	5.9 (5.3-6.5)
Not a carer with no chronic condition			1.3 (0.6-2.0)
Carer with chronic condition	22.2 (9.1-35.2)	23.0 (20.1-25.9)	11.8 (8.3-15.3)
Carer with no chronic condition			3.5 (1.7-5.3)
**Time spent caring**			
Carer with chronic condition	47.0 (33.1-60.9)	36.0 (24.9-47.1)	30.5 (20.6-40.4)
Carer with no chronic condition			41.5 (10.4-72.6)
**Time spent on health – self and caring**			
Carer with chronic condition	87.5 (68.8-106.2)	63.2 (46.1-80.2)	54.75 (38.9-70.6)
Carer with no chronic condition			76.5 (33.1-119.9)

1 Note: includes all values (i.e. includes zeros) but excludes exercise time.

2. Cells based on samples of less than 10 observations are not reported.

The median time spent on the HRA aspects of caring for another person ranged from around 30 hours per month (an hour per day on average) for the NSA sub-sample to around 47 hours per month (an hour and a half per day) for the Diabetes sub-sample. When combined with the time spent managing their own health the total time spent monthly on HRA by respondents ranged from 55 hours per month to 87.5 hours per month (equivalent to 2–3 hours per day) across the various groups.

### Time use of carer respondents within the highest decile

The times reported above, of between two and three hours a day spent on HRA, are not trivial amounts. However, at the 90^th^ percentile, the total of personal and caring HRA time is vastly greater at 248.5 hours per month for the Diabetes sub-sample, 313.0 for the Lung sub-sample and 294.0 for the NSA sub-sample (or between about eight and 10 hours a day).

### Relation of time use and number of chronic illnesses

The amount of time spent on HRA was associated with increasing number of carer chronic illnesses, reaching a peak with the very high time use by carers with five or more chronic illnesses. As shown in Table 4, the median time spent on HRA by carers with five or more chronic illnesses ranged from 68.3 hours per month in the NSA sub-sample to 113.7 hours per month in the Diabetes sub-sample (or almost 4 hours per day). Table 4 also shows that, as would be expected, the time spent providing care for people with five or more chronic illnesses is also high and is of a similar order to the total time spent by carers with large numbers of illnesses.

**Table 4:** Median total time spent related to health by respondent (hours per month)

	**Diabetes sub-sample**	**Lung sub-sample**	**NSA sub-sample**
**Respondents with five or more chronic diseases**			
Carers (own time plus caring time)	113.7 (81.7-145.7)	99.5 (69.1-129.8)	68.3 (24.3-112.2)
Non-carers (own time)	15.7 (9.8-21.6)	23.9 (16.6-31.2)	21.5 (15.6-27.4)
**Care recipients with five or more chronic diseases**			
Carers (own time plus caring time)	119 (76.8-161.2)	97.5 (75.0-120.0)	83.2 (23.3-143.0)
**Comparative numbers from overall respondents**			
Carer with chronic condition (own time plus caring time)	87.5 (68.8-106.2)	63.2 (46.1-80.2)	54.75 (38.9-70.6)
Non- Carer with chronic condition (own time)	10.5 (8.9-12.1)	15.7 (14.1-17.2)	5.9 (5.3-6.5)

While our study did not use disability measures we did observe that the number of chronic illnesses a person had was strongly correlated with the time their carer spent on HRA (with correlations 0.58, 0.58, 0.54 for the Diabetes, Lung and NSA sub-samples respectively; p = 0.00 in all cases).

### Time use for specific activities

As stated above, time use questions were segmented into HRA carried out every day, less frequent non-clinical activities, and those related to accessing clinical services. Attachment 1 (in the linked data file) shows details of HRA for each sub-sample and each category of time use. While the estimated time use differs between sub-samples, the overall patterns are very similar. Table 5 show the broad patterns for the Diabetes sub-sample as an example.

**Table 5:** Summary of caring and non-caring time spent on HRA activities for the Diabetes sub-sample only

	**Non Carer**		**Carer**					
**Time component**	**Time spent on self**		**Time spent on self**		**Time spent caring**		**Total time caring and on self**	
	**Percent responding**	**Median response**	**Percent responding**	**Median response**	**Percent responding**	**Median response**	**Percent responding**	**Median response**
	**372 respondents**	**Hours per month**	**55 respondents**	**Hours per month**	**55 respondents**	**Hours per month**	**55 respondents**	**Hours per month**
Total daily activities excluding exercise	91.9%	7.5	86.7%	17.5	83.8%	35.0	100.0%	61.0
Total daily activities including exercise	93.7%	20.0	90.4%	32.5				
Total general activities	81.6%	0.8	75.5%	2.0	89.5%	9.0	96.3%	7.0
Total medical activities	82.4%	2.0	82.8%	4.3	85.4%	6.5	94.8%	10.6
Overall total excluding exercise	95.5%	11.0	92.0%	25.0	100.0%	47.0	100.0%	87.5
Overall total including exercise	95.5%	26.0	95.7%	39.0			100.0%	88.8

• Daily activities include sorting/preparing/taking medications, carrying out treatments, testing and monitoring health, preparing special food and exercising or stretching.

• General activities include shopping for health items, shopping for special foods, attending rehabilitation, attending health education, attending support groups, and reading health information.

• “Medical activities” include organising appointments, organising travel to appointments, time in waiting rooms, attending consultations, blood tests/x-rays etc. and attending for other treatments.

Everyday activities took up most time spent on respondents’ own health care and on caring. The largest component of this was preparing special foods in the cases where this was required, which was relatively infrequent for respondents who were not carers (8-16%). However, between 32% and 48% of those caring reported preparing special foods and spent at the median between 30 and 60 hours per month on this activity (varying between the sub-samples). Shopping for health requirements (for example, medicines) is the most common of the non-clinical activities although the overall median time including caring and self-management activities is in the order of two hours per month in all sub-samples. Other activities like attending rehabilitation can be very demanding of time, but only applied to a small number of people. Across all sub-samples the most common clinically-related activity reported was sitting in waiting rooms, and this was the most time consuming or second most time consuming activity after travel depending on the sub-sample.

## Discussion

To our knowledge, our study is the first to measure time spent on specific HRA by informal carers who themselves have a chronic illness. The median time spent on health related caring activities of between 30.5 and 47 hours per month is comparable with median times estimated by Bittman et al. [23] of one hour and 27 minutes of care daily (45 hours per month). Their estimates included all care (including care of people with disabilities) rather than only health related care. Their study encompassed carers of all ages rather than the older cohort we have studied.

Informal carers did engage in numerous HRA to manage their own health and to assist others in the management of theirs. Some activities were undertaken by most informal carers, for example dealing with medications. More time consuming activities such as preparing special foods and carrying out treatments were carried out by fewer respondents.

The outstanding finding from these results is that informal carers with a chronic illness spent more time on their own HRA than respondents who were not informal carers, and when this was combined with their time caring for others, spent between four and nine times as long on HRA as non-carers. Those in the highest decile spent on average between eight and 10 hours each day on HRA.

We also identified a pattern of increasing time spent on HRA as the carer’s number of chronic illnesses increased. This pattern was also associated with the number of chronic illnesses of the care recipient. While increased morbidity was associated with increased time use, informal carers with chronic illnesses spent more time caring for others than engaging in activities associated with their own health care. The number of chronic illnesses a person had was strongly correlated with the time their carer spent on HRA. It is likely that the care recipient’s level of disability would also increase with their number of illnesses. Within the Diabetes sub-sample the reported prevalence of chronic pain and depression/anxiety was almost twice as higher for carers (48.1% and 42.8% respectively) than non-carers (25.3% and 20.4% respectively). We have reported the prevalence of chronic illnesses among survey respondents in another article, where self-reported rates of chronic pain and depression/anxiety were lower across the three sub-samples [33].

### Implications of findings for carer support strategies: allowances, respite and targeted services

In most industrialised countries the desire to minimise unnecessary hospital admissions and costly residential care is driving policies to support older people living within their communities, increasing reliance on the ‘unpaid workforce’ of carers [34]. Dejonge et al. [5], for example, have suggested a model of chronic illness management that reduces costs by shifting care into the community. However, while the model they propose might improve aspects of both the patient and carer experience, it pays no attention to the negative impacts of community-based care on carers.

Sustainable and effective informal care requires “centralised information dissemination, improved care coordination, merged funding streams, and expanded consumer direction” [17]. However, these policy goals also require a more nuanced understanding of the situation and actual capacities of carers. Our findings suggest that existing faith in the ongoing availability and capacity of informal care may be misplaced. Many carers face time demands which appear manageable, for healthy carers with limited other demands on their time, at between two and three hours a day. However, other carers managing their own chronic illnesses (s) and diminishing functional abilities as well as providing care to another are likely to be struggling. How, though, are policymakers to decide what changes are needed and to whom they should be targeted for best outcomes?

Welfare systems have utilised three key strategies to support informal carers; carer allowances, respite care and targeted service provision. In Australia, for example, carer allowances and respite care are part of the social security system, with home and community care programs generally managed within the healthcare system. Findings from this study suggest much of the burden of care problem cannot be solved simply by providing income supplements (although these would help) unless these are adequate to buy resources that reduce time demands on carers, and those resources are available. Minor increases in income support are unlikely to assist in better management of medications, transport to health-related appointments, allow access to adequate respite services or improve availability of affordable special foods. Some of the highest pressure points come from level of the carer’s own health, especially those with five or more chronic diseases. There is no simple way to identify this group and target additional resources to assist them.

To what extent should respite services for informal carers be part of future solutions? The Decima report from Canada suggests respite is imperative for caregivers [35]. Studies, both in Australia and internationally, have indicated barriers to the short-term use of respite services such as carers being unable to access services when they need them, and users feeling guilty about taking respite [3,36]. While the cost effectiveness of respite services remains under debate [37] the need for informal carers to support people with chronic illness is evident, and as other studies have shown, so too is their need for respite [3]. The high presence of chronic illness in the informal care population suggests even more urgent attention to addressing barriers of respite use is warranted. Future research to inform design of respite services should explore whether carer chronic illnesses are an additional barrier to respite use.

Furthermore, early work by Valderas and colleagues suggests that some combinations of illnesses may have characteristics in terms of time use and functional impairment that could lead to a better understanding of the needs of both carers and the people they care for [38-40]. Multi-morbidity research of carer populations could lead to better targeting of respite and other forms of support for people with multiple illnesses.

This study shows that some HRAs lead to the much higher demands on time, providing a focus to improve targeting of services. For example, while not relevant to all carers, preparing special foods and carrying out treatments are tasks associated with large blocks of time. In the case of preparing special foods one obvious option to reduce time burdens seems to be better and more focused utilisation of services of the style of “Meals on Wheels”. In Australia, Meals on Wheels (a non-government service run by volunteers) provides affordable meal preparation and delivery services for people who need it. People eligible for this service include those who are housebound, frail adults, people with a disability or illness and their carers. People with special dietary requirements and chewing or swallowing problems are catered for.

Strategies for enhancing Meals on Wheels-type solutions could move in two directions; expanding the range of meals to meet special dietary needs posed by multi-morbidity; and developing systems which overcome some of the complexities of current temporal arrangements. Regarding this second option, to meet recommended health safety targets meals must be prepared within a particular time and delivered within a particular time, with consumers (and often carers) at home and ready to consume or refrigerate meals when they arrive [41,42]. While the program currently does free up carers from preparing foods, it cannot offer them complete time flexibility (which has been raised as a point of frustration by carers in our previous qualitative research [42]). Strategies that move in these two directions could make significant reductions on time demands of some carers.

Like the preparation of special food, clinical treatments can be extremely time demanding for some groups of patients and carers. While some treatments cannot be safely or easily sped up, advances in ambulatory peritoneal dialysis, ‘satellite’ haemodialysis, and nocturnal dialysis treatments have significantly improved time use experiences of patients and carers by increasing the flexibility of when and where treatments can be undertaken [43,44]. The value of flexibility that nocturnal treatments offer to patients and carers’ waking lives cannot be over-stated, and may provide a model for consideration in relation to other time demanding treatments.

There are undoubtedly a wide range of measures available to simplify tasks and reduce time demands on carers. In the Australian context, for example, Dose Administration Aids (DOAs) such as Webster blister packs for the delivery of pharmaceuticals significantly reduce the amount of time people spend on sorting medications, but are not covered by the publicly funded Pharmaceutical Benefits Scheme. A subsidy would reduce cost barriers to the wider use of DOAs. The survey also found that failure to co-ordinate medical appointments leads to repeated travel and increased waiting times for carers. Virtual appointments and case conferencing have been identified as potential avenues for improving care coordination, however in the Australian setting up take has been low and the challenges posed by rural and remote locations have been considerable. Further efforts to better co-ordinate care are required.

Finally, an awareness of this pressing time burden strengthens arguments for constant critical appraisal of the efficacy for standard – often time consuming – self-management tasks. Research by Henderson and colleagues, for example, has identified that for people with diabetes, testing blood glucose levels may not be the most effective use of their time as they do not correlate with improved health outcomes [45].

### Limitations

This study is based on a relatively small number of survey respondents, from a survey with a 24% response rate. However, while time estimates vary between the sub-samples, broad patterns are similar. While we have shown that the time allocation to HRA varies according to the number of chronic illnesses experienced by carers, it also likely to depend on the health and functional ability of care recipients, how long carers or their care recipients have had a particular illness, the type of illness (of carer or care recipient), and/or the extent to which a care recipient can access support from others.

While we have discussed the findings in terms of health service options for improving support to carers, we acknowledge that in this survey we did not ask respondents to provide information on what kinds of support they wanted. However, we did ask this of participants in a previous qualitative study [28], and their responses have helped shape our discussion of health service support options.

### Suggestions for future research

As noted above, we suggest further research is warranted concerning carer multi-morbidity and barriers to respite use, including addressing other factors that influence time spent on HRA and the cost of that time to carers. This study has not developed the wider implications of caring, for example the impact of caring on personal health or the quality and duration of sleep [8,13] or ‘weathering’ associated with high physical, emotional and mental demands [46]. Nor has it addressed other temporalities associated with care such as process time, which references the multiple and interacting processes at play at a given time, that influence for example, the way we perceive and measure time spent caring [23,42]. The survey did not measure activities forgone due to caring and self-care responsibilities. Research addressing that would provide insight into the true ‘cost’ of time spent caring. The authors suggest that future research should link time spent on HRA with these kinds of factors to deepen our understanding of the actual ‘work’ of informal care.

## Conclusions

This paper set out to understand the time use of informal carers who themselves have a chronic illness. Many health policies and service programs assume that the informal carer is healthy and capable of caring for the care recipient. There is also an assumption held in society more generally that family members will be in a position to care for their loved ones and that they will willingly do so. In this study most informal carers were spouses or other nuclear family members. However, in this study most carers also had chronic illness themselves, and those with greater numbers of chronic illnesses were those who faced the greatest overall time demands, of between 8.5 and 10 hours a day devoted to HRA.

If carers are to be able to continue to provide support this suggests that programs focused on reducing the most severe time demands will be needed. Policy and health care services will need to adapt to recognise and reduce the time burden on those carers and their households. More carefully targeted investment in the social infrastructure of formal care would free up carers for other activities (including their own care) and holds the potential to improve the quality of life as well as the health outcomes of this population.

## Competing interests

The authors declare no competing interests. The funding organisation (NHMRC) had no role in the study design, data collection, analysis and interpretation, or the writing and publication of this article.

## Authors’ contributions

All authors made substantial contributions to conception and design, acquisition of data, primary analysis and interpretation of data; and were involved in drafting the manuscript and revising it critically for important intellectual content. Additionally, LY conceived of the study; IM and MB were heavily involved in primary data analysis; and TJ was heavily involved in drafting and revising the manuscript. All authors read and approved the final version of the manuscript.

## Authors’ information

This research was undertaken by three members of the Serious and Continuing Illness Policy and Practice Study at the Australian National University. The authors have training in anthropology, psychology and health services research. TJ is undertaking postdoctoral research at the Australian National University concerning experiences of time and chronic illness and this paper forms part of her research.

## Pre-publication history

The pre-publication history for this paper can be accessed here:

http://www.biomedcentral.com/1471-2458/13/374/prepub
